# Acute Exercise Improves Inhibitory Control but Not Error Detection in Male Violent Perpetrators: An ERPs Study With the Emotional Stop Signal Task

**DOI:** 10.3389/fnhum.2022.796180

**Published:** 2022-04-13

**Authors:** Chia-Chuan Yu, Chiao-Yun Chen, Neil G. Muggleton, Cheng-Hung Ko, Suyen Liu

**Affiliations:** ^1^Department of Kinesiology and Health Education, The University of Texas at Austin, Austin, TX, United States; ^2^Department of Athletic Sports, National Chung Cheng University, Minxiong, Taiwan; ^3^Department and Graduate Institute of Criminology, National Chung Cheng University, Minxiong, Taiwan; ^4^Institute of Cognitive Neuroscience, National Central University, Taoyuan City, Taiwan; ^5^Brain Research Center, National Central University, Taoyuan City, Taiwan; ^6^Institute of Cognitive Neuroscience, University College London, London, United Kingdom; ^7^Department of Psychology, Goldsmiths, University of London, London, United Kingdom; ^8^Integrated Drug Addiction Treatment Center of the Jianan Psychiatric Center, Ministry of Health and Welfare in Taiwan, Taipei, Taiwan

**Keywords:** violence, executive function, International Affective Picture System (IAPS), event-related potential (ERP), aerobic exercise

## Abstract

Violence has been linked to the co-occurrence of cognitive dysfunction and altered activations in several brain regions. Empirical evidence demonstrated the benefits of acute exercise on motor inhibition and error detection and their neuronal processing. However, whether such effects also hold for the population with violent behaviors remains unknown. This study examined the effects of acute aerobic exercise on inhibitory control and error monitoring among violent offenders. Fifteen male violent offenders were counterbalanced into experimental protocols, which comprised a 30-min moderately aerobic exercise [60% heart rate (HR) reserve] and a 30-min reading control session. After each session, participants performed an emotional stop signal task while event-related potentials (ERPs) were recorded simultaneously. Results showed insignificant changes in ERPs components [i.e., N2, P3, error-related negativity (ERN), and error-positivity (Pe) amplitudes] and the behavioral performance in go condition, stop accuracy, and post-error adjustments by exercise. However, the current study demonstrated that the acute exercise facilitated stop signal reaction time (SSRT) when compared to the control session regardless of emotional conditions. This is the first research to exhibit the improvements in inhibitory performance by acute exercise for violent offenders. Most importantly, this effect was independent of affective settings, expanding the existing knowledge of the influences of acute exercise on cognition. Our findings implicate the perspective of acute exercise for clinical and correctional practices.

## Introduction

Violence is one of the leading issues that affects countries and incurs considerable social costs (Soares, 2006). The worldwide economic impact of violence was more than 14 trillion dollars in 2017 (Iqbal et al., 2019), thus addressing this severe problem is warranted. This behavioral outcome is also associated with other adverse consequences in health, such as sexual risk behaviors (Raj et al., 2006), human immunodeficiency virus (HIV) infection (Sareen et al., 2009), illicit drug use (Menard et al., 2015), alcohol dependence/abuse (Anne Lown and Vega, 2001), and poor health status (Hurwitz et al., 2006; Stewart et al., 2012), all of which result in decreased wellbeing and numerous burdens in public health. Despite interventions that have been identified for treating violent behaviors and their recidivism, such as those based on the Duluth model or cognitive-behavioral therapy, the effect sizes varied (Babcock et al., 2004). In addition, such treatments require tremendous resources and funding for implementation, suggesting the necessity to develop a cost-effective alternative for those with violent behaviors.

Violent behavior has been characterized by impulsivity (Zhou et al., 2014) and emotional dysregulation (Garofalo and Velotti, 2017). From the perspective of cognitive neuroscience, research suggests that failures in motor inhibition and the neural processing subserving in it may present the core cognitive deficits underlying the manifestation of violent behavior or aggression (Vilà-Balló et al., 2014; Brugman et al., 2016; Qiao et al., 2016; Wallinius et al., 2019). Inhibitory control is one of the elements in executive function, which refers to the capacity to withdraw proponent behaviors and suppress irrelevant information (May and Hasher, 1998) that plays a vital role in development and learning (Carlson and Moses, 2001; Handley et al., 2004; Eggers et al., 2013; Jaekel et al., 2016). Neuropsychological studies have demonstrated the reduction in the allocation of attentional resources for inhibitory control and prolonged stop signal reaction time (SSRT) (Vilà-Balló et al., 2014) and lack of error detection and post-error adjustment (Chen et al., 2014) among violent offenders. Furthermore, neuroimaging data reported that violent individuals or those with higher aggressive traits exhibited structural and functional aberrations in brain regions that are important for inhibition, error detection, and emotional regulation, such as the amygdala, dorsolateral and dorsomedial prefrontal cortex, and the anterior cingulate cortex (Pardini and Phillips, 2010; Schiffer et al., 2011; Prehn et al., 2013; Leutgeb et al., 2016), which might be the potential mechanisms that account for their aberrant cognitive and behavioral performance.

The non-invasive approach of event-related potentials (ERPs) provides insights into cortical activations that respond to external stimuli and cognitive processing with a high temporal resolution. N2 and P3 components of ERPs were frequently investigated in the tasks that require response inhibition; the N2 amplitude was proposed to reflect the processing of conflicting information (Donkers and Van Boxtel, 2004; Groom and Cragg, 2015) and the activity from the anterior cingulate cortex (Van Veen and Carter, 2002), while the P3 amplitude was thought to represent the inhibition of planned actions and the allocation of attentional resources that are commonly observed in the activities in frontoparietal areas (Smith et al., 2007; Nguyen et al., 2016; Waller et al., 2019). Researchers have observed decreased P3 and N2 among juvenile and adult violent perpetrators in the combined flanker/stop signal task (Chen et al., 2014; Vilà-Balló et al., 2014). Similarly, these studies demonstrated reduced error-related negativity (ERN) and error-positivity (Pe) in violent offenders than in controls, which are the two ERP components that could be measured after the onset of an incorrect response, as being the index of error detection by ERN, and reflect error recognition and error-related adjustment *via* Pe (Nieuwenhuis et al., 2001).

Interestingly, a growing body of evidence suggests that a single bout of exercise intervention benefits cognition and emotional state by affecting their neuronal processing in several brain regions (Basso and Suzuki, 2017). Within a wide range of literature, acute exercise has been examined for its effects on inhibitory control with increased accuracy, shorter reaction time (RT), shorter SSRT, and/or altered N2 and P3 amplitudes in a variety of tasks that require motor inhibition among different populations (Joyce et al., 2009; Chu et al., 2015; Wang et al., 2015; Netz et al., 2016; Kao et al., 2017; Ligeza et al., 2018). Further, it is suggested that acute exercise produces disproportionate benefits on inhibition at both behavioral and neuroelectric levels. However, findings in the effects of acute exercise on error monitoring were inconclusive.

Pontifex et al. (2013) found that following a moderately acute exercise, children with attention-deficit/hyperactivity disorder (ADHD) demonstrated increases in accuracy and post-error slowing (PES) but not RT in a modified flanker task. Although the ADHD group had a smaller ERN amplitude when compared with the healthy matched controls in the reading session, their ERN amplitudes did not differ after exercise. Implicating that acute exercise might improve the underlying processing of error detection and the behavior of post-error adjustment. Whereas others indicated no influence of acute exercise on the indices of error monitoring (Larson et al., 2015). Instead, some scholars suggested that this aspect of cognition may be more susceptible to fitness performance (Themanson and Hillman, 2006). Perhaps the discrepancies in samples (i.e., individuals with and without neurodevelopmental disorders), study designs, and exercise intensities utilized in previous studies lead to this divergence. Nevertheless, the relationship between acute exercise and error monitoring has received less attention than other cognitive functions, such as inhibition and memory.

To the best of our knowledge, no study has been conducted to investigate whether an acute aerobic exercise could improve cognitive performance and influence neuroelectric activities among violent offenders. Therefore, the present study aimed to determine, for the first time, whether a 30-min acute aerobic exercise with a moderate intensity affects inhibitory control and error monitoring among violent offenders. Given the inconsistent findings of error monitoring, it is worthy of examining whether other facets should also be controlled when probing the effects of acute exercise. Emotion is one of the commonly manipulated variables in the error monitoring literature due to its importance and modulation role for error-related brain activities (Wiswede et al., 2009; Hobson et al., 2014). Despite extensive evidence showing improved emotion after acute exercise (Basso and Suzuki, 2017), it is still unclear whether the benefits of exercise on cognitive function hold for varied affective settings. In light of this, we employed a modified stop signal task with negative and neutral emotional stimuli, by which to elicit affective responses while the ERPs were recorded simultaneously to examine if the effects of acute exercise vary between emotional conditions. Based on the literature, we hypothesized that a brief aerobic exercise session would improve inhibition by decreasing the SSRT (i.e., shorter SSRT) and greater cortical activations for inhibition and conflict information processing (i.e., larger N2 and P3 amplitudes) in both emotional conditions. Enhanced error monitoring was also predicted where the acute exercise was given, with prolonged PES, increased post-error accuracy (PEA), and enhanced ERN and Pe amplitudes regardless of emotional settings. However, a recent meta-analysis (Ishihara et al., 2021) focused on the moderation of cognitive demand showed that the benefits of acute exercise on executive function are more considerable when a higher cognitive demand is introduced and in consideration of presenting emotional images in which the stop signal task could interfere both cortical activities and behavioral performance in executive control (Verbruggen and De Houwer, 2007; Camfield et al., 2018), which should require more cognitive resources than the neutral emotion, we anticipated that the effects of acute exercise on dependent variables to be greater in the presence of negative stimuli than in the neutral condition. To best represent the populations with violent behaviors, we recruited individuals who had a history of violent offense(s) (e.g., homicides and bodily harm) that was linked to decreased executive control in literature.

## Materials and Methods

### Participants

Twenty male volunteers were recruited from two correctional facilities affiliated with the Agency of Corrections, Ministry of Justice in Taiwan (Tainan prison: *n* = 10; Taipei prison: *n* = 10). They were selected based on their official and self-reported crime history and had been convicted for a violent offense(s) against victims (*M*_*sentenced incarceration*_: 177.30 months, SD: 85.85), such as attempted homicide, homicide, or wounding. Taking into account the influences of substance use on cognition and/or brain activities (O’Leary et al., 2000; Weissenborn and Duka, 2003; Marshall et al., 2007; Strand et al., 2019) and different forms of violence may reflect distinct personality and offense characteristics (Gudjonsson and Sigurdsson, 2000; Craig et al., 2006; Swogger et al., 2007), we only included the participants whose offense(s) was occurred without any influences of alcohol or drugs and had not committed domestic violence or sexual crimes. Participants were excluded from this study if any of the following inclusion criteria was not met: (1) without a history of neurological or psychological disorder; (2) without a history of brain injury; (3) free from physiological and cardiovascular illness; (4) had a normal or corrected-to-normal vision; (5) right-handed; and (6) without any medical or physical conditions that impeded them from performing exercise activity, assessed by the Physical Activity Readiness Questionnaire (PAR-Q). Five individuals were excluded from analysis due to the inability to understand the instruction, history of brain injury, having no history of violent crime, quitting during the study, and/or poor quality of ERPs data. The final analysis included 15 participants (*M*_*age*_: 30.13 years, SD: 7.95).

### Study Design and Procedure

Due to the policies in prisons and the difficulty of recruiting participants for the current study, we encountered the limitation of sampling in prisons. Therefore, we were not able to conduct a true experimental study. Instead, the present study was implemented with a counterbalance design to examine the effects of acute exercise while eliminating the potential order effect. The interventional orders were randomly assigned with that 50% of participants performed the exercise intervention first while the other half of participants conducted the reading session first. This experiment took place in the classrooms in the prisons mentioned earlier.

Each participant visited the study location on 3 separate days with no more than 7 days apart, and they were told not to engage in any forms of rigorous exercise on the days of the visit. After debriefing the study aim and procedure at the first visit, participants completed the consent form, questionnaires, and the resting heart rate (HR) measurement. For the second and third visits, participants were counterbalanced into the interventional sessions, which were 30 min of either seated reading sport- and health-related books or aerobic exercise with moderate intensity on a stationary cycle ergometer (Monark Ergomedic 828E, Sweden) with the resistance set at 2.5–3.0 kp. To ensure that participants were actively engaged in reading, they were required to briefly summarize the content following the reading session. Before the exercise intervention, participants conducted a 10-min stretching to prevent exercise injury. The acute exercise session included a 5-min warm-up, which started with a speed of 80 beats per min, a 20-min main exercise at the intensity of 60% heart rate reserve (HRR) (American College of Sports Medicine, 2013; Sandroff et al., 2015), and a 5-min period of cool-down cycling. The exercise intensity was maintained by adjusting the resistance and speed of cycling to control the HR and the rating of perceived exertion (RPE), which were measured by a Polar FT7 HR Monitor (Polar Electro Inc., Finland) and the Borg RPE scale (Borg, 1982) respectively in every minute throughout the exercise session. The RPE scale was designed to measure the perception of the exercise intensity and has been reported strongly correlated to HR (*r* = 0.74) (Scherr et al., 2013). The score ranges from 6 (very, very light) to 20 (very, very hard). Our goal was to keep the score between 12 and 15 so that participants would subjectively perceive as receiving a moderately intense exercise.

After each session, there was an approximate 5-min period for setting up the ERP recording instruments. Participants then completed the cognitive assessment while the neuroelectric activities were recorded concurrently. Each participant received NT$500 as a reimbursement after completing the experiment (or received a portion of this amount if the experiment was not completed). All experimental procedures were initiated after participants signed the written consent form. The current study was approved by the Human Research Ethics Committee at Chung Cheng University and was implemented according to the Declaration of Helsinki.

### Measurements

#### Reactive-Proactive Questionnaire

The aggressive tendency among participants was determined by the Reactive-Proactive Questionnaire (RPQ), which is a 23-item questionnaire that consists of reactive aggression (11 items) and proactive aggression (12 items) subscales with great validity and reliability (Cima et al., 2013; Dinić et al., 2020). Participants were requested to choose the occurrence frequency (from 0 represents never to 2 represents often) with items, such as “gotten angry when others threatened you” (reactive) or “used force to obtain money or things from others” (proactive).

#### International Physical Activity Questionnaire-Long Form (IPAQ Long-Version)

The self-administrated Taiwanese version of IPAQ (long-version) is a tool with good content validity for estimating physical activity (Liou et al., 2008), participants were asked to recall the frequency and the quantity of time that they spent for each category (i.e., vigorous, moderate, and walking) of physical activity in the past 7 days, then the metabolic equivalent in minutes per week (MET-min/week) was calculated to reflect the energy consumption.

#### Fluid Intelligence

Participants administered the widely studied Raven’s Advanced Progressive Matrices (APM), which is a test that provides a non-verbal estimation of fluid intelligence by two sets of the test (set I: 12 items; set II: 36 items) with great reliability and validity across countries (Bors and Stokes, 1998; Rushton et al., 2004; Nurhudaya, et al., 2019; Khairani et al., 2020). Set I was used as a practice material while set II served as a formal measure, the score ranges from 0 to 36 with the higher score reflecting better intelligence level. However, due to the limited time, we were allowed to visit prisons, we had to reduce the number of items in set I. Thus only the first to fourth questions of the collection I were kept in practice.

#### Cognitive Assessment

**FIGURE 1:**
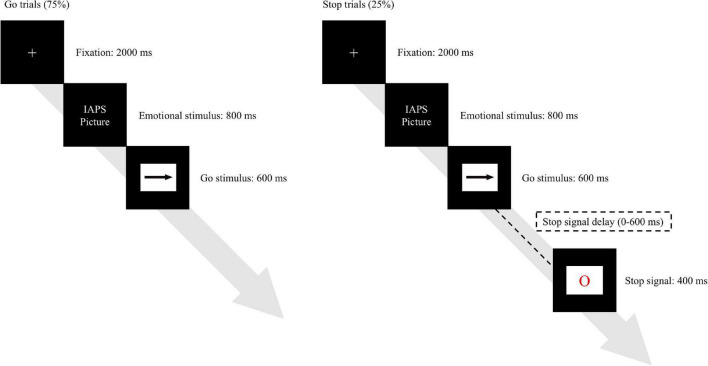
The procedure of emotional stop signal task.

An emotional stop signal task (Figure 1) was presented on a laptop using E-Prime 2.0 software (Psychology Software Tools, Pittsburgh, PA, United States; Schneider et al., 2012) with participants seated comfortably 57 cm apart from the monitor while the center of the screen was set at about eye level. All stimuli were presented on a white background; the task firstly presented a fixation cross (2,000 ms) followed by an emotional stimulus (800 ms), then a go target (an arrow pointed to either left or right for 600 ms or until a reaction was made) was introduced where a response should be made with the correct reaction being to point out its direction *via* pressing “D” or “L” key on keyboard using left or right index finger when seeing a left- or right-pointing target. Participants were instructed to respond to the go target as quickly and accurately as possible. To induce the motor inhibition processing, we employed the stop signal with the probability that random 25% of the go stimuli were accompanied by a stop signal (a red circle lasted for 400 ms). The initial time interval between the onsets of a go stimulus and a stop signal (i.e., stop signal delay, SSD) was set as 200 ms, and a successful inhibition was defined as withdrawing the response to the go stimulus. The SSD would be adjusted based on the performance of the previous stop trial to produce a stop accuracy rate at about 50%; if participants failed to withhold a go response, the SSD would be reduced by 34 ms for the subsequent stop trial, whereas a success inhibition led to adding 34 ms in the SSD of the next stop trial, the range of SSD was limited between 0 and 600 ms. A reminder “Too slow! Please respond faster…” written in Mandarin on a black background would appear for any miss or reactions slower than 600 ms in go trials.

The emotional stimuli were adopted from the International Affective Picture System (IAPS), which has been broadly used in emotion studies and has shown its effectiveness of evoking emotional reactivity with great consistency (Gong and Wang, 2016). Sixty pleasant and sixty unpleasant emotion pictures were chosen from the IAPS (see “Supplementary Material S1” for details). This task contained eighty trials of six blocks (three neutral and three negative blocks, total 480 trials) with equal probabilities for the go stimuli pointing left and right, each block comprised of 20 emotional stimuli with each emotional stimulus was randomly repeated 4 times. To avoid the order effect for emotional arousal, we adopted a counterbalanced design that 50% of the participants performed negative blocks first while others conducted the neutral trials first. Moreover, the orders for presenting the blocks in each emotional condition were randomized with a 5-min break between the blocks. An emotional free practice block of 80 trials with the same procedure was conducted before the formal assessment to ensure that all participants were acquainted with the instruction.

The analyses for behavioral performance were conducted for both negative and neutral conditions as follows: *(1) go accuracy (%)*: the proportion of correct go responses among all go trials; *(2) go RT (ms)*: the mean RT for correct go responses; *(3) stop accuracy (%)*: the proportion of successful performance among all stop trials; *(4) SSRT (ms)*: an index that reflects the time for inhibitory processing, which was calculated according to the race model (Logan and Cowan, 1984) and the distribution of go RTs along with the probability of successful inhibition for different SSDs (Hanes et al., 1998; Hanes and Carpenter, 1999); *(5) PES (ms)*: this was obtained by deducting the “mean go RT that immediately followed a correct go trial” from the “average go RT that immediately followed an incorrect stop trial”; and (6) *PEA (%)*: the PEA was calculated by deducting the “average go accuracy that immediately followed a correct go trial” from the “mean go accuracy that immediately followed an incorrect stop trial.”

#### Electroencephalographic Activity Recording and Processing

Electroencephalographic (EEG) data were collected using NuAmps amplifier in combination with Scan 4.5 software from 32 scalp electrodes mounted on an elastic electrode cap (32-channel Quick-Cap, Compumedics Neuroscan, Charlotte, NC, United States) according to the International 10–20 System (Homan, 1988; Klem et al., 1999). The electrooculographic (EOG) activity was monitored by the electrodes that were placed above and below the left orbit and at the outer canthus of both eyes, with the electrodes at both sites of the mastoid (A1 and A2) serving as reference points. Our data were measured with the electrode impedances below 5 kΩ and a sampling rate of 500 Hz while the AC recording and a 0–100 Hz bandpass filter were applied.

The offline ERPs data processing was conducted using Scan 4.5 software. For stimulus-locked mean amplitudes, epochs were extracted from −100 to 800 ms relative to the onset of each stop signal where the successful inhibition occurred, whereas the epochs for response-locked average amplitudes were set as −200 to 1,000 ms surrounding the response to a stop signal. A finite impulse response (FIR) with low-pass zero-phase filtering (30 Hz; 12 dB/octave), baseline correction (−100 to 0 ms for N2 and P3, −200 to 0 ms for ERN and Pe), artifact rejection (trials with the signal that exceeded ± 100 μV were rejected), and an ocular artifact correction that was based on the algorithm developed by Semlitsch et al. (1986) were performed for each participant and then all trials were finally averaged for negative and neutral conditions separately.

Through the visual inspection of ERP patterns and according to the exercise and cognitive control literature, the Cz site was chosen for analyzing stimulus-locked ERPs (Kok et al., 2004; Wang et al., 2015), with N2 was defined as the waveform within a 170–300 ms time window after the onset of the stop signal, while P3 was evaluated as in the range of 310–410 ms following the onset of the stop signal. The mean amplitude for ERN, in contrast, was defined as the negative deflection of EEG activity at FCz (Falkenstein et al., 1991, 2000; Themanson and Hillman, 2006; Moreau et al., 2020) within a window of 80–280 ms relative to the onset of response, and the Pe was defined as the positive deflection of EEG at Pz (Falkenstein et al., 2000; Themanson and Hillman, 2006; Moreau et al., 2020) within a range from 480 to 780 ms relative to the response. The average numbers of artifact-free trials for stimulus-locked ERPs following exercise were 30.60 (negative emotion; SD: 2.29) and 29.27 (neutral emotion; SD: 2.52), while after reading were 29.87 (negative emotion; SD: 2.07) and 29.87 (neutral emotion; SD: 2.17). Similarly, the mean amounts of artifact-free trials for response-locked components in the exercise session were 28.47 (negative emotion; SD: 3.42) and 28.00 (neutral emotion; SD: 5.08), while in the reading session were 29.00 (negative emotion; SD: 2.54) and 28.87 (neutral emotion; SD: 2.36), which are sufficient for analyses (Rietdijk et al., 2014).

### Statistical Analysis

Statistical analyses were performed using IBM SPSS Statistics 26.0 with the significance level set at α = 0.05. Descriptive statistics were analyzed for age, body mass index (BMI), fluid intelligence score, RPQ ratings, and physical activity. Furthermore, we conducted two-way repeated-measures analyses of variance (ANOVAs) with a 2 (interventional sessions) × 2 (emotional conditions) design for comparing behavioral performance and ERPs mean amplitudes. A Bonferroni approach was used for follow-up comparisons if any significant main effects or interactions were observed, effect sizes (partial eta-squared, η_*p*_^2^) along with observed power and 95% confidence intervals (CIs) are provided for significant results.

## Results

### Demographics and Questionnaires

Mean and SD for demographics and questionnaires are shown in Table 1. Our participants were young to middle Asian male adults (*M*_*age*_: 30.13 years, SD: 7.95) with lower educational attainment (*M*_*education*_: 10.27 years, SD: 1.62) and acceptable to obese BMI (*M*_*BMI*_: 25.39, SD: 3.61). Possibly due to the living environment and regulations in prisons, their amounts of physical activity range from very low to medium (*M*_*MET/week*_: 5836.23, SD: 8184.21). Although participants have been selected based on their criminal history, here we also conducted additional independent *t*-tests to compare the aggressive tendency (i.e., RPQ scores) between the current sample and an age-matched non-violent offender group that is from another dataset in our lab (also see details in “Supplementary Material S2”). The *t*-tests showed that the proactive aggression [*t*(20.06) = 3.61, *p* < 0.01, 95% CI (0.97, 3.61)], reactive regression [*t*(30) = 2.78, *p* < 0.01, 95% CI (0.65, 4.26)], and summed RPQ score [*t*(30) = 3.42, *p* < 0.01, 95% CI (1.91, 7.57)] were all higher for the violent offenders than for the non-violent offenders.

**TABLE 1:** Descriptive statistics of demographic and questionnaire data.

Variables	*M* (SD)
Age (years)	30.13 (7.95)
Education attainment (years)	10.27 (1.62)
BMI (kg/m^2^)	25.39 (3.61)
Fluid intelligence score	13.27 (5.30)
RPQ: Proactive subscale	2.93 (2.22)
RPQ: Reactive subscale	5.87 (2.70)
RPQ: Summed score	8.80 (4.66)
Physical activity (MET-min/week)	5836.23 (8184.21)

### Behavioral Performance

**FIGURE 2:**
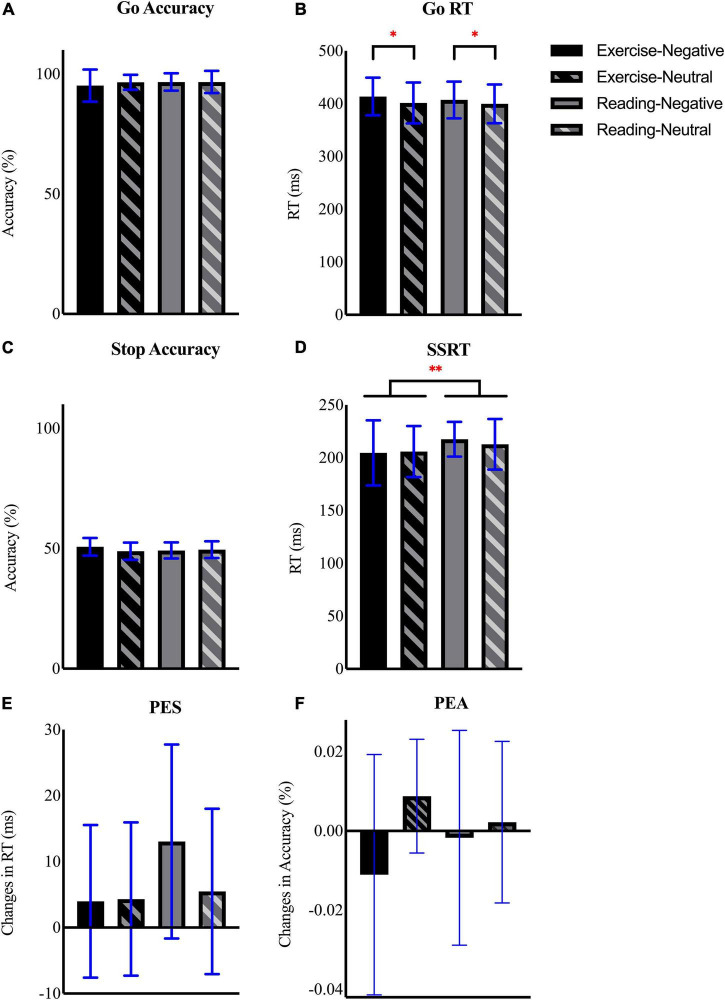
Mean and SD of **(A)** go accuracy, **(B)** go reaction time (RT), **(C)** stop accuracy, **(D)** stop signal reaction time (SSRT), **(E)** post-error slowing (PES), and **(F)** post-error accuracy (PEA) across interventional sessions and emotional conditions. *Refers to *p* ≤ 0.05, ^**^refers to *p* ≤ 0.01.

All behavioral data are shown in Figure 2. The repeated measures ANOVA showed no significant main effect or interaction for the go accuracy (all *p*s > 0.05; Figure 2A). Although the results demonstrated no main effect of intervention (*p* = 0.66) and interaction for go RT (*p* = 0.53), we found a significant effect of emotion [*F*(1,14) = 7.77, *p* = 0.02, η_*p*_^2^ = 0.36, observed power = 0.74] with that the negative emotion induced a slower go RT when compared with the neutral condition [*p* = 0.02, 95% CI (2.25, 17.28); Figure 2B].

The analysis for stop accuracy showed neither main effect nor interaction (all *p*s > 0.05; Figure 2C). Similarly, analysis of SSRT revealed insignificant main effect of emotion (*p* = 0.66) and interaction (*p* = 0.35). However, there was a main effect of session on SSRT [*F*(1,14) = 5.10, *p* = 0.04, η_*p*_^2^ = 0.27, observed power = 0.56], follow-up comparison exhibited a shorter SSRT after exercise than after reading [*p* = 0.04, 95% CI (−19.31,−0.50); Figure 2D]. For both PES (Figure 2E) and PEA (Figure 2F), the repeated measures ANOVA showed neither main effect nor interaction between factors (all *p*s > 0.05). To further examine whether this effect varied with emotional images, we performed an additional paired sample *t*-test to compare the differences in SSRT between interventional sessions (i.e., exercise-reading; ΔSSRT) among two emotional conditions. The result showed no significant difference in ΔSSRT between negative and neutral conditions [*t*(14) = − 0.97, *p* = 0.35, 95% CI (−19.56, 7.41)].

### Event-Related Potentials Outcomes

**FIGURE 3:**
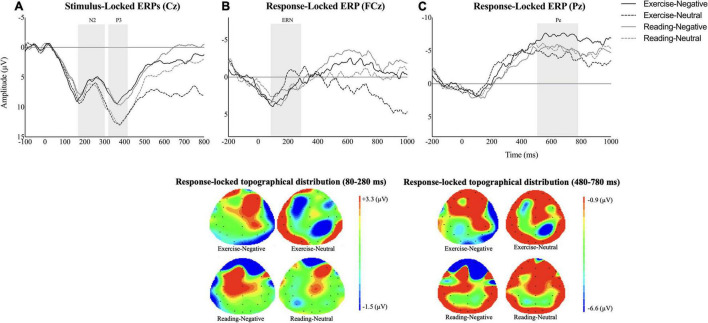
Average waveforms across interventional sessions and emotional conditions for **(A)** stimulus-locked event-related potentials (ERPs) at Cz (N2: 170–300 ms; P3: 310–410 ms), **(B)** error-related negativity (ERN) at FCz (80–280 ms), and **(C)** Pe at Pz (480–780 ms). The additional scalp topographies are also provided to depict the response-locked activities within time widows of 80–280 ms and 480–780 ms across conditions.

Event-related potential data are depicted in Figure 3. Analysis showed a significant main effect of emotional stimuli on P3 amplitude [*F*(1,14) = 8.64, *p* = 0.01, η_*p*_^2^ = 0.38, observed power = 0.78] with a decreased P3 waveform for the negative condition than for the neutral emotion [*p* = 0.01, 95% CI (−5.78,−0.90)]. Nevertheless, the ANOVAs demonstrated neither main effect of session/emotion nor interaction between independent variables for N2, ERN, and Pe (all *p*s > 0.05).

## Discussion

The fact that acute exercise can lead to enhanced cognitive control is our primary interest; however, whether such benefit also holds for the population who behave violently is unknown. Given that violent offenders were described as impulsive (Zhou et al., 2014) and deficient in inhibitory control (Vilà-Balló et al., 2014; Brugman et al., 2016; Qiao et al., 2016) and error monitoring (Chen et al., 2014), the findings of this study could be valuable for clinical and correctional settings. We recruited prisoners who committed the extreme offense(s), such as murder to include the representative sample. Unsurprisingly, these violent individuals had greater RPQ scores, reflecting the personalities that put them at higher risks for violent crime and repeated offenses (Cima et al., 2013; Zijlmans et al., 2021). The present study provides initial evidence that one brief bout of aerobic exercise with moderate intensity may be an effective intervention for individuals with violent behaviors. Using both behavioral performance and neurophysiological measures to evaluate the influences of a single 30-min acute exercise on fundamental components of cognition, our data clearly indicated improvement in the behavior of inhibitory control instead of error monitoring. Furthermore, by utilizing the emotional stop signal task, we determined if the benefits of acute exercise occur in different affective conditions. As expected, the manipulation *via* the IAPS stimuli produced evidently affective reactivities with that our sample showed a longer go RT together with a declined P3 amplitude when negative pictures were presented. This observation is in line with the literature, which demonstrated that visual stimuli with a negative emotion could interfere with behavioral performance (Verbruggen and De Houwer, 2007) and the allocation of attentional resources manifested by P3 amplitude at frontocentral sites (Verona et al., 2012).

The behavioral data exhibited a shorter SSRT after acute exercise than following the reading session, reflecting the increased ability of inhibitory control induced by our intervention. Interestingly, the exercise-induced behavioral change was only for stop trails rather than go performance, suggesting the disproportionate effect of acute exercise for improving inhibition but not simple reaction choice (Chu et al., 2015). Our findings supported the notion that the amount of time for successfully administering the process of motor response inhibition could be facilitated by one single bout session of moderate exercise, which is consistent with previous studies that examine populations with or without cognitive deficits, such as methamphetamine addicts and healthy young adults using varied tasks that demand inhibition (Chu et al., 2015; Wang et al., 2015), reflecting the benefits of acute exercise on motor inhibition across populations. In accordance with the horse-race model proposed by Logan and Cowan (1984), the two separate processes of *go* and *stop* are independent and *race* with each other. The competition between these two systems determines the behavioral outcome in a given task; if the *go* process finishes before the *stop* process, a response to the stop signal will be more likely to occur; contrariwise, a successful inhibition will be achieved if the *stop* process finishes ahead of the *go* reaction. Therefore, the authors argue that our observation of the more efficient inhibition instead of the general reaction performance could be attributed to the facilitation role of acute exercise on the *stop* but not the *go* process. Moreover, the analysis of ΔSSRT contravened our hypothesis of the larger benefit of acute exercise during the presence of negative emotion. The increased inhibition appeared in not only neutral but also negative conditions, which extends the current understanding of the effects of acute exercise on emotion and motor inhibition that were often examined separately. Since emotional difficulties (Gillespie et al., 2018) and suffering from events that produce negative moods (e.g., violent and bullying victimization) (Aceves and Cookston, 2007; Reed et al., 2015) might be risk factors for aggressive behaviors, this exciting finding has implications for future treatment program designs. In contrast, the two behavioral adjustments that come after errors, the PES and PEA, were somewhat stable throughout the experimental protocols. Although the results contradicted our second hypothesis, they are consistent with the research focusing on non-offender adults. Among the limited studies that investigated the effect of acute exercise on PES, only one indicated that acute exercise evoked a greater PES, yet this effect was observed in ADHD children but not in healthy controls (Pontifex et al., 2013), whereas others showed the null effect of acute exercise on PES for adults (Themanson and Hillman, 2006; Davranche and McMorris, 2009; Larson et al., 2015). On the other hand, previous studies exhibited that the indices of error action detection could be influenced by cardiorespiratory fitness (Themanson and Hillman, 2006; Themanson et al., 2008) and the amount of physical activity (Themanson et al., 2006), which were not examined by the current study. Thus, further examination of these effects on violent offenders is warranted.

Event-related potentials have been extensively adopted to provide an insight into how the brain functioning responded to inhibitory control when an acute exercise was introduced (Themanson and Hillman, 2006; Chang et al., 2012, 2017; Chu et al., 2015; Aguirre-Loaiza et al., 2019). Specifically, the N2 and P3 amplitudes serve as neurophysiological markers of inhibition that probe the underlying mechanisms of exercise-induced behavioral change. The neuroimaging literature demonstrated that in addition to the behavioral effects, acute exercise also enhanced N2 (Ligeza et al., 2018) and P3 components (Chang et al., 2017), which correspond to the optical imaging data that revealed greater oxygen-hemoglobin in the left dorsolateral prefrontal cortex after acute exercise among healthy adults (Yanagisawa et al., 2010). In spite of what was found in the literature, the data only partially supported our first hypothesis that also predicted increased N2 and P3 amplitudes for the exercise session. Unlike the majority of previous exercise and ERPs studies, the modulations of N2 and P3 by exercise were not observed. This lack of changes might be associated with the task design that we used. Although the IAPS stimuli are helpful for investigating the behavioral and neuroelectric effects under different affective settings, the increases in emotional arousal could bring in the alteration in cognitive processing in general, which may further blunt the effects of exercise. To disentangle the effects of exercise on emotion and inhibition, the adoption of other hybrid task designs for future ERPs studies is encouraged. Additionally, the post-error ERP signatures also revealed no effects of acute exercise. To increase the compatibility between desired outcomes and actual performance, one must consistently monitor, compare, and regulate ongoing behaviors. This internal process involves two different components – the *evaluative* and *executive* mechanisms – that are associated with the activities in the anterior cingulate cortex and dorsolateral prefrontal cortex (Van Veen and Carter, 2006), while the ERN and Pe amplitudes may reflect these two mechanisms, respectively (Hajcak et al., 2005; Overbeek et al., 2005). Our findings are, again, similar to the previous exercise and action monitoring studies that showed invariant ERN and Pe by acute exercise (Themanson and Hillman, 2006), suggesting that a single bout of moderate aerobic exercise may not be capable of increasing the *evaluative* or *executive* elements of error processing among violent individuals.

## Limitation and Future Direction

There are a few limitations in the current study. First, we could neither measure the dependent variables in the follow-up multiple time points nor employ the gold standard of randomized controlled trial due to the characteristics of our sample and the research environment. The ERPs and behavioral results could partially result from the repeated exposure to the task materials. However, participants were counterbalanced into each experimental protocol and emotional condition to eliminate the potential order effect. Secondly, because the research locations were not able to be rigorously controlled as usually described in previous studies (e.g., light- or sound-attenuated laboratories), this confounding factor could make noise in our data. Third, with the small sample size, our data should be treated as exploratory, and one must be cautious when interpreting these findings as they may not be generalizable to larger violent populations. Furthermore, the lack of a healthy-matched control group led to the inability to explore whether the effects of acute exercise are different between violent and non-violent adults. For future directions, the comparison between released offenders and healthy controls is warranted to understand the effect of acute exercise in post-incarceration settings. Furthermore, we encourage investigating the effects of acute exercise and exploring which settings these influences are more prominent. For instance, the manipulation of awareness of error and the parameters of cognitive assessment could be considered simultaneously since the presence of behavioral adjustment is dependent on the awareness of error (Klein et al., 2007) and response-stimulus interval (Buzzell et al., 2017), which are the factors that we did not scrutinize. Considering the crucial role of emotion and feedback on neuroelectric responses of inhibition and error monitoring, future studies could explore if the benefits of exercise occur when different categories of emotion (e.g., sadness, aversion, happiness, and anger) and implicit or explicit feedback are given. Finally, the ERN and Pe components were not studied with the same rigor as other ERPs (e.g., N2 and P3) in exercise studies, hence, we suggest future works study the relationship between exercise and error detection and adjustment in combination with other neuroimaging techniques with higher spatial resolution, such as functional magnetic resonance imaging (fMRI) and functional near-infrared spectroscopy (fNIRS) to give a more comprehensive understanding.

## Conclusion

The present study demonstrates novel findings that support the notion that moderately acute aerobic exercise facilitates inhibitory control among violent offenders. Specifically, the results assessed utilizing the emotional stop signal task suggested that the acute exercise facilitated the *stop* process instead of the *go* process, and this benefit is independent of emotional conditions. However, the effects of an acute exercise intervention on error monitoring variables are not available in the current study, which replicated the findings in previous studies, which showed that *evaluative* and *executive* mechanisms of error monitoring are not susceptible to acute exercise among adults. Furthermore, the insignificant ERP findings that leave the neurological explanation remain unclear. Therefore, further studies will be needed to examine the underlying mechanisms of the benefits of acute exercise for violent individuals by recruiting a larger sample size along with matched controls and assessing the effects in the follow-up time points after acute exercise. Our preliminary evidence warrants future studies to examine the potential role of acute exercise in treating violent behavior in other clinical or sub-clinical populations.

## Data Availability Statement

The raw data supporting the conclusions of this article will be made available by the authors, without undue reservation.

## Ethics Statement

The studies involving human participants were reviewed and approved by the Human Research Ethics Committee at Chung Cheng University. The participants provided their written informed consent to participate in this study.

## Author Contributions

C-CY: conceptualization, methodology, formal analysis, investigation, data curation, writing – original draft, writing – review and editing, and visualization. C-YC: conceptualization, methodology, resources, data curation, supervision, and funding acquisition. NM: methodology, software, and resources. C-HK: formal analysis. SL: conceptualization, methodology, data curation, writing – review and editing, supervision, and funding acquisition. All authors contributed to the article and approved the submitted version.

## Conflict of Interest

The authors declare that the research was conducted in the absence of any commercial or financial relationships that could be construed as a potential conflict of interest.

## Publisher’s Note

All claims expressed in this article are solely those of the authors and do not necessarily represent those of their affiliated organizations, or those of the publisher, the editors and the reviewers. Any product that may be evaluated in this article, or claim that may be made by its manufacturer, is not guaranteed or endorsed by the publisher.
