# Supplementary material for: Acute Exercise Improves Inhibitory Control but Not Error Detection in Male Violent Perpetrators: An ERPs Study With the Emotional Stop Signal Task
Source: Front Hum Neurosci. 2022 Apr 13;16:796180. doi: 10.3389/fnhum.2022.796180 (PMC9045000; doi:10.3389/fnhum.2022.796180)
Supplement: Supplementary file 1 [file Data_Sheet_1.pdf]

Supplementary Materials

S1. List of emotional stimuli

The current study used the following emotional stimuli from the International Affective Picture System (IAPS) for the emotional stop signal task. The description of the task design and procedure can be found in the Methods section of the main text.

Type		Number of stimuli
Pleasant	Set 1	1440, 1450, 1460, 1463, 1500, 1510, 1540, 1590, 1600, 1602, 1603, 1604, 1610, 1620, 1660, 1670, 1710, 1731, 1750, 1810
	Set 2	1812, 1850, 1900, 1910, 1920, 1942, 1947, 5000, 5001, 5010, 5020, 5030, 5200, 5201, 5220, 5250, 5260, 5270, 5300, 5390
	Set 3	5480, 5593, 5594, 5660, 5700, 5711, 5720, 5740, 5750, 5760, 5779, 5780, 5800, 5811, 5820, 5870, 5890, 7009, 7233, 7270
Unpleasant	Set 1	2055.1, 2352.2, 3000, 3010, 3030, 3060, 3061, 3064, 3069, 3071, 3101, 3102, 3120, 3130, 3140, 3150, 3160, 3168, 3266, 9400
	Set 2	9405, 9420, 9433, 3005.1, 3022, 2095, 2900, 3181, 3220, 3230, 3301, 3350, 9530, 9040, 9050, 9342, 9410, 9415, 9440, 9471
	Set 3	9480, 9635.1, 9910, 9920, 2683, 3500, 3530, 6211, 6244, 6250, 6312, 6313, 6510, 6550, 6570, 6571, 6831, 6838, 9252, 9265

S2. Comparisons of the Reactive-Proactive Questionnaire (RPQ) scores between the current sample and an age-matched non-violent offender group.

In addition to using criminal history as an inclusion criterion for our participants, we performed additional independent *t*-tests to examine whether the participants in the current study have higher aggressive traits than an age-matched non-violent offender group from another dataset in our lab. These non-violent offenders (n = 17) were recruited from the same prisons as our violent individuals. However, they were sentenced due to the offense(s) other than violence (e.g., forging instruments or seals, larceny, or fraudulent). The *t*-tests showed that the proactive aggression (t (20.06) = 3.61, $p < .01$, 95% CI [.97, 3.61]), reactive regression (t (30) = 2.78, $p < .01$, 95% CI [.65, 4.26]), and summed RPQ score (t (30) = 3.42, $p < .01$, 95% CI [1.91, 7.57]) were all higher for the violent offenders than for the non-violent offenders.

Table S2 Comparisons of the Reactive-Proactive Questionnaire (RPQ) scores between the current sample and an age-matched non-violent offender group

Variables	Violent group (n = 15) M (SD)	Non-violent group (n = 17) M (SD)	t (df)	p
Age	30.13 (7.95)	31.06 (6.94)	-.35 (30)	.73
RPQ: Proactive subscale	2.93 (2.22)	.65 (1.12)	3.61 (20.06)	.002
RPQ: Reactive subscale	5.87 (2.70)	3.41 (2.29)	2.78 (30)	.009
RPQ: Summed score	8.80 (4.66)	4.06 (3.11)	3.42 (30)	.002
